# Reconstruction of cropland cover changes in the Shandong Province over the past 300 years

**DOI:** 10.1038/srep13642

**Published:** 2015-09-16

**Authors:** Yu Ye, Xueqiong Wei, Fan Li, Xiuqi Fang

**Affiliations:** 1 School of Geography, Beijing Normal University, Beijing 100875, China; 2 Key Laboratory of Environment Change and Natural Disaster, Ministry of Education, BNU, Beijing 100875, China

## Abstract

To advance global change rssearch, it is essential to reconstruct changes in historical cropland coverage on a regional scale in China. This paper presents data collected from 244 local gazetteers, government statistical records and remote-sensing land cover data from the Shandong Province. The study reconstructed the spatial distribution of the rate of reclaimed land at the county level and compared this map with a map of the current spatial distribution of suitable cropland. The following conclusions were drawn: (i) The rate of cultivated land grew exponentially. The extent of reconstruction in cropland areas during the 17^th^ century, 18^th^~19^th^ centuries, the beginning of the 20^th^ century, the 1980 s, and the beginning of the 20^th^ century are 4.51 mha, 6.51 mha, 7.52 mha, 8.53 mha and 11.80–12.00 mha, respectively. (ii) Several agricultural centers formed during the late 17^th^ century. Until the beginning of the 20^th^ century, the reclamation rate increased rapidly near the four southern lakes, which are located in the Zaozhuang and Linyi regions. (iii) Most reclamation activities before the 19^th^ century occurred in suitable agricultural areas, and the cultivated land was already reclaimed by the beginning of the 20^th^ century.

Reconstruction of historical land cover changes has progressed significantly over the past 20 years with the establishment of regional and global land cover data sets^1^,^2^,^3^,^4^,^5^. For Chinese historical land cover reconstruction, scholars often use the part of China that is included in the Global Land Use Database (termed the SAGE data set), which was established by the Center for Sustainability and the Global Environment at the University of Wisconsin–Madison^2^, the Historical Database of the Global Environment (termed HYDE data sets), which was produced by the Netherlands Environmental Assessment Agency^6^, and the PJ datasets, which were established by the Planck Institute of Meteorology in Germany^4^.

However, the accuracy of these global datasets when applied at regional scales is uncertain. Zhang *et al.*^7^ compared the reconstruction of Chinese cropland areas in traditional agricultural areas during the middle of the 17^th^ century with the portion of China that is included in the aforementioned global data sets. The cultivation ratios in the global HYDE and PJ data sets appear to be lower than those in the Chinese regional data sets, which are based on historical cropland records. Comparing regional reconstructions of cropland cover in northeastern China over the past 300 years (termed CNEC data sets) and historical data from the Scandinavian Peninsula with the two global land cover data sets, the following conclusions can be drawn: the trends of the data sets and the phase characteristics exhibit differences, and there are clear deviations in the spatial distribution of cropland in the global land cover data^8^. Because of inaccuracies and regional deviations in the global data sets, the results, when applied to Chinese regional climate modeling, often deviate from reality^9^,^10^.

Reconstruction of Chinese regional land cover to advance global change research is necessary, and will fulfill any impending needs in research regarding the impact of land use/cover changes and climate modeling. The accuracy, resolution and accessibility of historical land cover data sets are key issues. At present, a reconstruction of cropland cover in traditional Chinese agricultural areas, with uniform spatial and temporal resolutions, is necessary for national-scale research. These data are sourced from historical documents and are validated using data-calibration methods. The reconstruction results are also used to produce gridded data sets^10^,^11^,^12^. At the same time, many regional research studies in China show that long-term regional land cover changes are nonlinear and highly dynamic, and these changes are also the result of intensive interactions with the local social history^11^,^13^,^14^,^15^,^16^,^17^,^18^,^19^,^20^,^21^,^22^,^23^. The popular global-scale reconstruction method is not always suitable for reconstruction of Chinese regional land cover^24^. In China, there are still gaps in these regional-scale reconstructions. Therefore, it is important to strengthen the exploration of regional-scale reconstruction methods, establish a series of methods that are suitable for historical Chinese regional cropland cover reconstruction, and obtain regional cropland data sets. Reconstruction studies of China’s historical cropland cover have unique characteristics and other differences that distinguish them from Western countries. Reconstruction in China uses historical documents as the primary proxy data; these documents are tax unit records, not actual survey data, and contain more nonstandard descriptions than uniform statistical data.

We collected data from 244 county gazetteers in Shandong that were compiled during the Qing Dynasty and the Republic of China period as well as statistical data since 1916 that was gathered from the central government and modern remote-sensing land cover data. The historical document data were calibrated via a correlation analysis of data from different sources using trend substitution and spatial interpolation. We examined five time periods—the late 17^th^ century, the 18^th^–19^th^ centuries, the beginning of the 20^th^ century, the 1980 s, and the beginning of 21^st^ century—to reconstruct the cultivated land area at the county level in Shandong. Next, a spatial distribution map of the reclamation rate was produced. A modern-day map of the suitable cropland area was then compared with historical data to estimate the saturation level of land reclamation in different regions and during different periods. The contributions of this study are the following: (i) it explores applicable reconstruction methods to study the regional historical cropland cover change in China and to establish regional data sets; (ii) it provides data sets of historical cropland cover changes in Shandong that are based on the county administrative unit; and (iii) the findings are valuable for local land use planning and land policy formulation.

## Data Sources and Methods

### Research area

**Figure 1:**
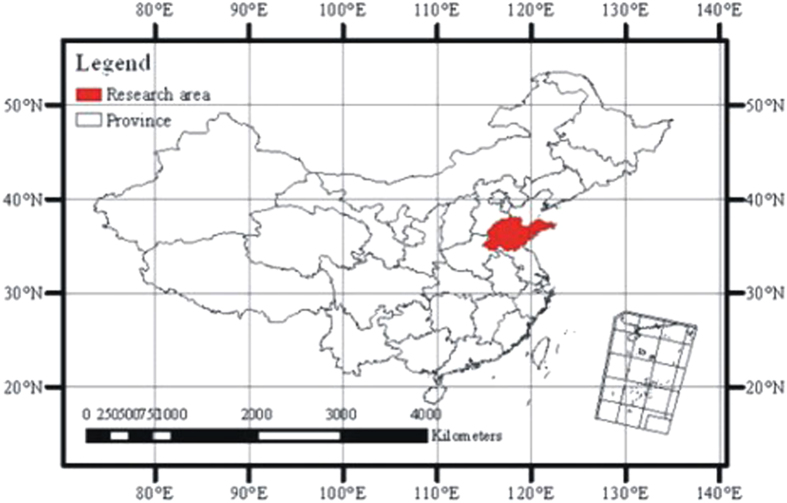
Location of research area (map created using ARCGIS).

**Figure 2:**
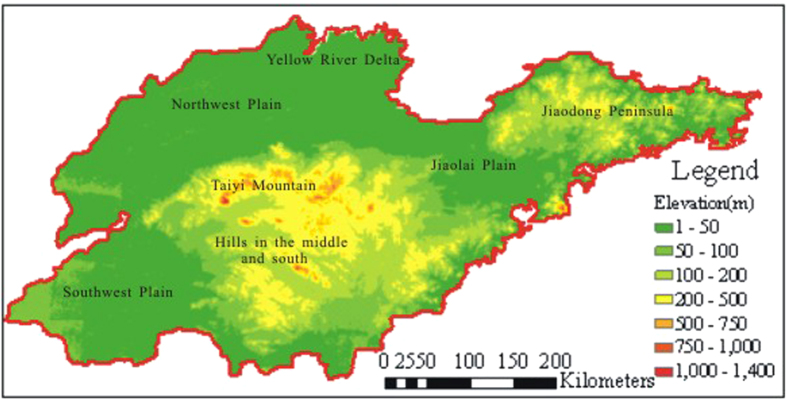
Topography and elevation of Shandong (1-km DEM) (map created using ARCGIS).

**Figure 3:**
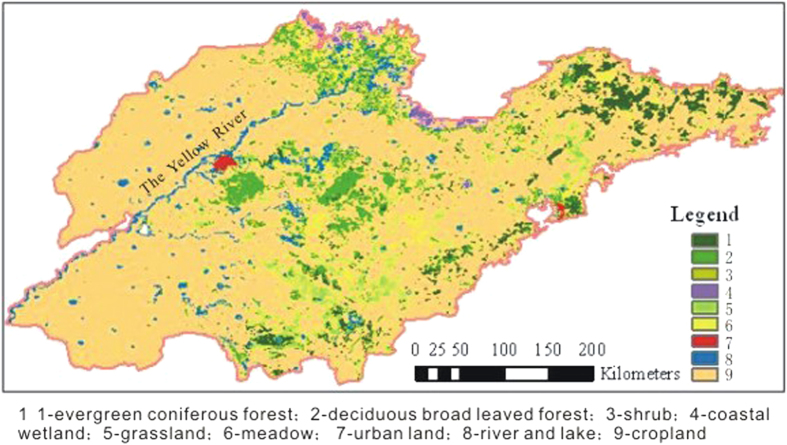
Land use and land cover during 2000 in Shandong (China/1-km resolution GLC2000) (map created using ARCGIS).

The research area for this study is the Shandong Province of China, which is located in the middle latitudes of the northern hemisphere, in the approximate range of N34°22′52″E114°19′53″–N38°15′02″E122°43′; the area includes 111 cities and counties. The area is located on the east coast of China on the lower reaches of the Yellow River; and is in the middle and northern sections of the Beijing-Hangzhou Grand Canal. The area is near the eastern shore of the Bohai and Yellow Sea and faces the Korean Peninsula and Japanese Islands across the sea. It is bordered by the Hebei Province to the northwest, the Henan Province to the southwest, and the Anhui Province and Jiangsu Province to the south (Fig. 1). The topography of Shandong is primarily plains or mountainous/hilly areas, which occupy 55% and 28.7% of the total land area, respectively. The mountain and hill areas are located in the middle and southern portion of Shandong. The Taiyi Mountain is at the center, and the geography transitions from low hills to a flood plain around the Yellow River. To the west and north of Shandong is the northwest alluvial plain area, which was formed by the Yellow River and is one part of the North China Plain. The East Peninsula consists mostly of rolling hills (Fig. 2). Shandong has a semi-humid monsoon climate and is in a warm temperate zone. The climate is mild, and four seasons are discernible. The annual average temperature for the entire province is 11 °C–14 °C. Its annual average precipitation ranges from 550 mm to 950 mm. Shandong is an important agricultural area in China. It has extensive croplands, located mainly in the northwestern plain, the southwestern plain, and the Jiaolai plain. Forests and grasslands are found in hilly areas that are located in the middle and to the south of Shandong, the Jiaodong Peninsula, and the Yellow River Delta. Wetlands are distributed along the coast (Fig. 3).

### Methods framework

**Figure 4:**
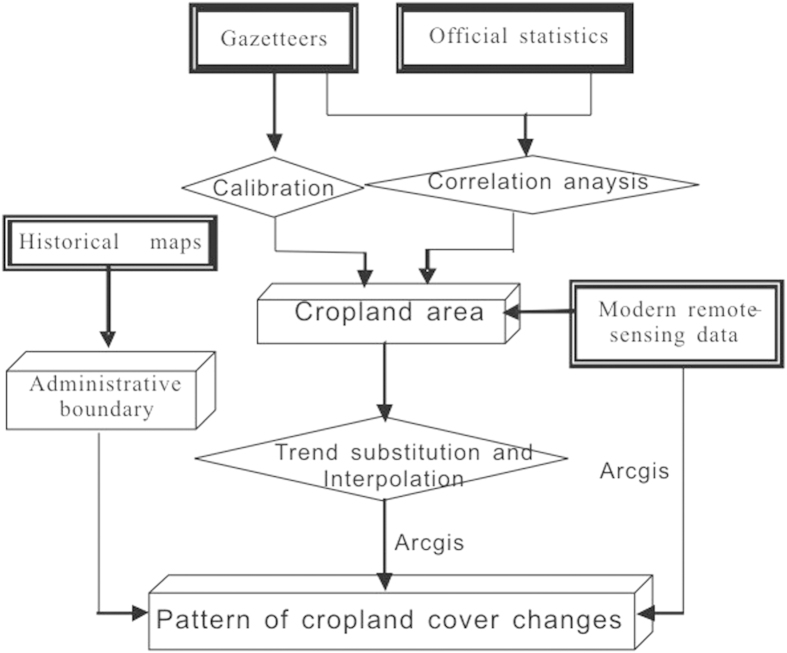
Technical route sketch of reconstruction of cropland cover changes.

In this study we used local gazetteers in Shandong, which were recorded during the Qing Dynasty and the Republic of China period; government statistics, and land cover data derived from modern remote sensing. Next, the historical document data were calibrated using a correlation analysis of the data from various sources and trend substitution and spatial interpolation to reconstruct the uniform cultivated land area at the county level in Shandong. i) A calibration method was used to transform the historical land tax descriptions into actual cropland area. ii) Using a correlation analysis of the calibrated cropland area data, which were sourced from gazetteers and statistics from the beginning of the 20^th^ century, enabled reconstruction of dependable estimates of the cropland area and cultivation ratios. iii) Trend substitution and spatial interpolation of the gazetteer cropland data were used to reconstruct the cultivation ratios for four time periods in the late part of the 17^th^ century, the 18^th^ century, the 19^th^ century and the beginning of the 20^th^ century. Finally, we used ARCGIS to produce a spatial distribution map of the reclamation rate at the county level and compared it with a modern-day map of the suitable cropland area to estimate the saturated land reclamation level in different regions and different periods. A sketch of the technical methods used in this paper is shown in Fig. 4.

### Calibration of historical data

#### Data from the Shandong gazetteers during the 17^th^–20^th^ centuries

Cropland data for the Qing Dynasty and the Republic of China period were gathered from 244 gazetteers associated with the Shandong Province; these data covered the entire research area, including 110 current cities and counties. Two to four versions of the gazetteers for each city or county were compiled during different periods. They recorded data for the cropland areas, and can be used for comparison and verification. Different types and grades of cropland data were recorded in the gazetteers of Shandong. The primary factors that affect the dependability of cropland records in the Shandong Province during the Qing Dynasty include the application of different land unit systems (for example, Mu and Qin), tax discounts, and social factors, which include exaggeration and concealment in the reports.

To calibrate data from historical documents, we adapted the original cropland area data in the gazetteers using the following steps. First, we summed the measurements for different types and grades of cropland area to obtain a total cropland area for each county. Second, we converted Qin and Mu units into a modern-day unit, ha; for example, 1 Qin = 100 standard Qing Mu, and 1 standard Qing Mu = 0.9216 Mu (at present) = (0.9216/1500) km^2^ = (0.9216/15) ha. Third, we used a calibration coefficient of 1.2 for the total cropland area described above for each county to compensate for deviations resulting from tax discounts^25^,^26^. Fourth, we used coefficients of 0.9 and 1.2 for before and after 1736, respectively, to calibrate the total cropland area and to compensate for exaggeration and cheating^25^,^26^. Finally, we obtained an estimate of the uniform and actual cropland area at the county level in the Shandong Province for the 17^th^–20^th^ centuries, which was consistent with the current data.

#### Statistical data from 1916 in the Republic of China

Besides the data in the gazetteers, we used cropland area data for Shandong that was compiled at the beginning of the 20^th^ century. These data came from the *Fifth Agricultural and Commercial Statistics Table*^27^, which contains statistical data from the Republic of China. The data set contains 106 data points, which cover all 101 cities and counties in Shandong. Using the same method of calibration used to calibrate the historical gazetteer data, we converted the statistical cropland area data into the actual cropland area.

### Correlation analysis of data from different sources

**Figure 5:**
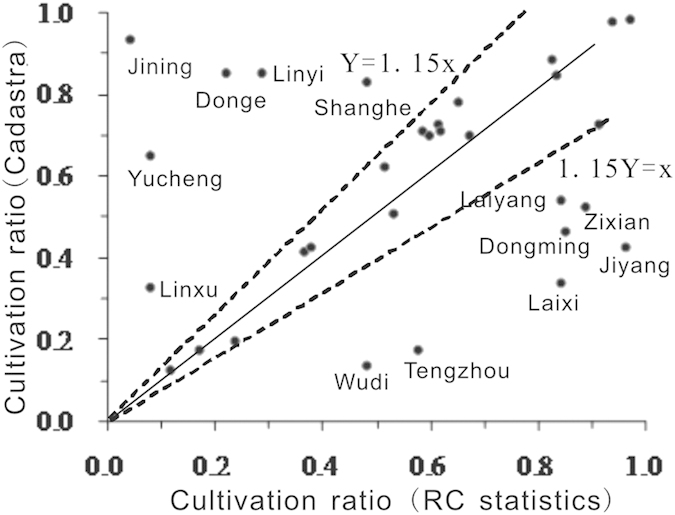
Comparison between calibrated data that were sourced from statistics from the Republic of China and calibrated data that were sourced from gazetteers at the beginning of the 20^th^ century (map created using Excel).

Based on the modern administrative base map, the cultivation ratio of each county at the beginning of the 20^th^ century was calculated using the calibrated cropland area data from gazetteers and statistics from the Republic of China. These two cultivation ratio data sources were overlaid and compared. Approximately 60% of the data in the 37 overlapping counties had coverage differences that were less than or equal to 15%, and data for six counties with cultivation ratios greater than one were removed (Fig. 5).

After comparing several versions of gazetteers and checking for administrative changes, we individually analyzed the data from 14 counties that exhibited differences greater than 15%. The statistical data from Jiyang, Dongming, Shenxian, Wudi, and Tengzhou in the Republic of China were evaluated prior to use because the data gathered from gazetteers used the previous version and are therefore lower than the actual values. The gazetteer data for Yucheng, Linqu, Dong’e, and Jining are more reliable than the data from the periods before and after, and the statistical data from the Republic of China are too low. The gazetteer data of Linyi, Laiyang, and Laixi should be applicable because they have been revised to account for administrative changes by comparing different versions. For Shanghe, there is no evidence that the data are unreasonable.

In addition to these points, the two sets of data show a strong correlation. The linear regression equation is Y = 1.11x, where R^2^ = 0.94. Gazetteer-sourced data show a greater amount of cropland area than the statistical data sets, which may capture inconsistencies between the time periods or an increase in the cropland area at the beginning of the Republic of China.

Prior to applying the gazetteer data, the beginning of 20^th^ century was treated as one time period because of the consistency of the data sources. For the overlaid part of the two data sets, unreasonable data were substituted with gazetteer data (Changyi, Weifang, Laiwu, Yucheng, Linqu, Dong’e, Jining, Linyi, Laiyang, Laixi), and all reasonable statistical data from the Republic of China for this time period were used. Then, a consistent series was constructed together with gazetteer data gathered from the other two sections during the Qing Dynasty.

### Trend substitution and spatial interpolation

From the gazetteers, we obtained 211 calibrated cropland area data points in one to four time periods for every county. The total number of data points in the late 17^th^ century (concentrated in the 1650 s–1690 s), the 18^th^ century, the 19^th^ century and the first half of the 20^th^ century (1900–1937) was 56, 57, 56, and 42, respectively.

**Figure 6:**
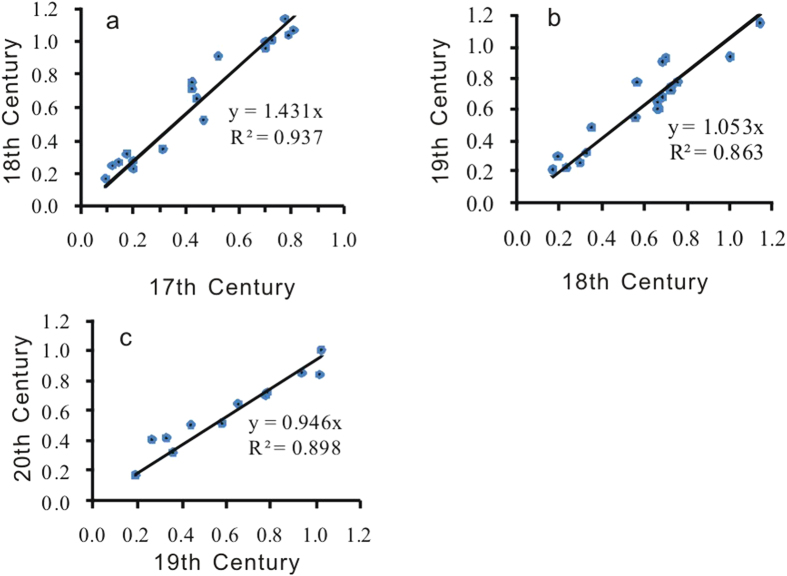
Correlation analysis of the superimposed data during four historical time periods (map created using Excel).

To compensate for administrative changes, gazetteer-sourced cropland data from the late part of the 17^th^ century, the 18^th^ century, the 19^th^ century and the beginning of the 20^th^ century were transferred to a modern administrative base map to calculate the cultivation ratio of every county. The historical cultivation ratio map reflects four time periods covering 47, 47, 46, and 37 counties. The cultivation ratios for the overlapping counties during these four time periods were correlated and analyzed (Fig. 6).

The analysis indicates a good linear correlation between the cultivation ratios of the 18^th^ century and the late 17^th^ century (Fig. 6a), between the 19^th^ century and the 18^th^ century (Fig. 6b), and between the first half of the 20^th^ century and the 19^th^ century (Fig. 6c), with an explained variance of 93.7%, 86.3%, and 90.0%, respectively (overlaid parts include 22, 21, and 13 points, where four, two, and one singular points were removed, respectively). According to the analysis of land reclamation trends in Shandong during the Qing Dynasty, fertile land cultivation reached its maximum extent, and reclamation reached a saturated, stable status that continued until the Yongzheng (1723–1735) and Kangxi Kingdoms (1736–1795)^25^. These results are also consistent with the coefficients in the regression equations of overlaid data for the four time periods.

Using trend substitution and interpolation, the spatial distribution of the cultivation ratios were reconstructed using two time periods during the Qing Dynasty, the late 17^th^ century and the 18^th^–19^th^ centuries. For the 18^th^–19^th^ century, we primarily applied the data from the 18^th^ century, and we interpolated using data from the 19^th^, 20^th^ and 17^th^ centuries, in that order of priority, by multiplying these data with the corresponding coefficients in the linear regression equation. In total, 86 data points from the 18^th^ and 19^th^ centuries were applied, which accounted for 81.9% of total cropland data for this time period. Additionally, for the late 17^th^ century time period, 47 data points from the late 17^th^ century were used. Others were interpolated using data from the 18^th^, 19^th^, and 20^th^ centuries, in that order, and they were multiplied by the corresponding coefficients of the linear regression equations. Data from the late 17^th^ century and 18^th^ century accounted for 80.7% of the total data.

### Spatial analysis of cropland cover changes

#### Calculation of cultivation ratios in the 1980 s and 2009

Shandong’s 1980s cropland cover data were gathered from a regional table of land use status for Shandong in the *Chinese National Land Resource Data Sets (Volume 3)*, which was compiled by the Committee of Comprehensive Investigation of Natural Resources, Chinese Academy of Science and State Planning Commission^28^. Cropland area and county area were retrieved from survey tables of land use in Shandong during the 1980 s. The cultivation ratio was equal to the cropland area divided by the county area for every county during that time period.

Cropland cover data for the beginning of 21^st^ century were sourced from a 300 m-resolution Global Land Cover Map (known as GlobCover 2009), which was created by the European Space Agency (ESA)^29^. The boundary of the modern-day Shandong Province was used to cut the Global Land Cover Map (GlobCover 2009) and attain a land cover map of the Shandong Province. Cropland cover types were extracted and summed to obtain cropland area data for every county in Shandong in 2009. Cropland area was divided by the land area in every county to calculate the cultivation ratio.

#### Analysis of cropland cover changes

Based on the reconstruction results described above and on studies of provincial cropland areas in Shandong during the Qing Dynasty and in modern times, we chose different time periods and used them to determine the extent and variation of the cropland areas during each time period; the purpose was to analyze the changing trends of the cropland area in Shandong over the past 300 years. We compared the reconstruction results of this paper with the results of Cao^26^ with a trend analysis of eastern China over the past 300 years by Ge^30^ and Liang^31^, and with an analysis of cropland extent changes since 1949 by Yu^32^ and Huang^33^.

Based on the above comparisons, we produced a spatial distribution map of cultivation ratios at the county level for five time periods: the late 17^th^ century, the 18^th^–19^th^ centuries, the beginning of the 20^th^ century, the late 20^th^ century and the beginning of the 21^st^ century. By subtracting these areas from each other, we obtained a map of cropland area changes. We compared this map with a 1-km gridded map of a suitable type of Chinese land (including cropland, forest, pasture, swamp and water) (Data Sharing Infrastructure of Earth System Science in China, www.geodata.cn), and we analyzed the relationship between them. We considered the impact of land reclamation, and estimated the saturation extent of land reclamation in different regions in Shandong over the past 300 years.

## Results and Analysis

### Trends in the Shandong cropland extent by time period over the past 300 years

**Figure 7:**
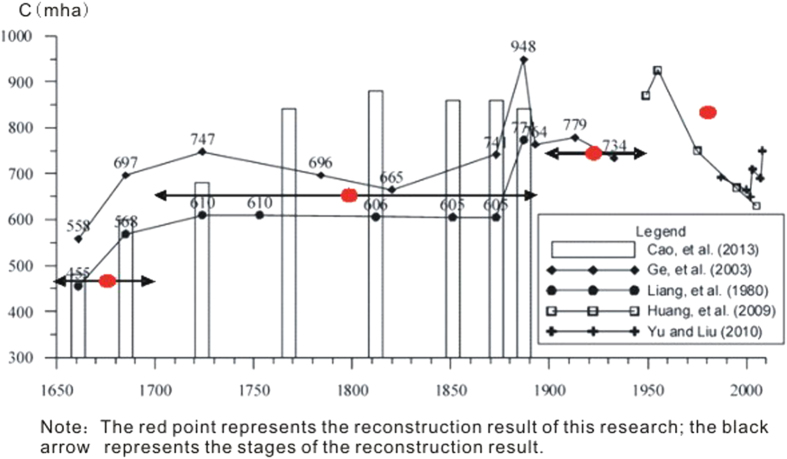
Trends in cropland area in Shandong (map created using Grapher).

According to published studies on the reconstruction of cropland data and a trend analysis of Shandong (Fig. 7), several stages were found. i) The first stage was the rapid-increase stage of the Shun-Kang-Yong Kingdom (1644–1735). The cropland area increased from 4.50–5.50 mha to 6.00–7.50 mha during this period, which is an increase of 1.50–2.00 mha. ii) The second stage was a stable stage of cropland area changes. The revised data compiled by Cao^26^ and Ge *et al.*^30^ showed that the cropland area changed by 6.50–8.50 mha, and there are different hypotheses about when the peak value appears and regarding the results of the changes that occurred during this stage. Cao’s^26^ revised results showed that the cropland area remained within 8.00–8.50 mha, a relatively high level, whereas the extremely high value that was recorded in 1887 does not exist. Ge *et al.*^30^ use the trend substitution method and substituted the 1952 data for the 1887 cropland area data from the late Qing Dynasty; this approach would probably increase estimates of the peak value of cropland area in Shandong. iii) The third stage consisted of a slightly decreasing stage during the late Guangxu Kingdom (1875–1908) and at the beginning of the Republic of China. The estimate by Ge *et al.*^30^ is within 7.00–8.00 mha. iv) The fourth stage was the period since the liberation of China. Cropland area appeared to reach a maximum value of 9.20 mha in the 1950 s, and then it gradually decreased. From 2001–2008, the cropland area fluctuated within 6.50–7.50 mha. The statistical data collected since the establishment of the People’s Republic of China in 1949 probably underestimated the cropland cover. Cropland cover area in Shandong was sourced from remote-sensing data in 2000, and in 2009, this area reached a maximum of 11.80–12.00 mha.

In this study, the total revised cropland area for every county in the late 17^th^ century, 18^th^–19^th^ centuries, and 1916 was 4.51 mha, 6.51 mha, and 7.52 mha, respectively. The cropland area in Shandong in the 1980 s was 8.53 mha^28^. These results are relatively consistent with the conclusions described above regarding the trends and stages of croplands in Shandong. The county data described above in different time periods, together with the 2009 data, are sufficient to illustrate the five stages of cropland area changes, the extent of the croplands and any regional differences during the relatively stable periods.

### Changes in the spatial distribution of cropland cover in Shandong over the past 300 years

**Figure 8:**
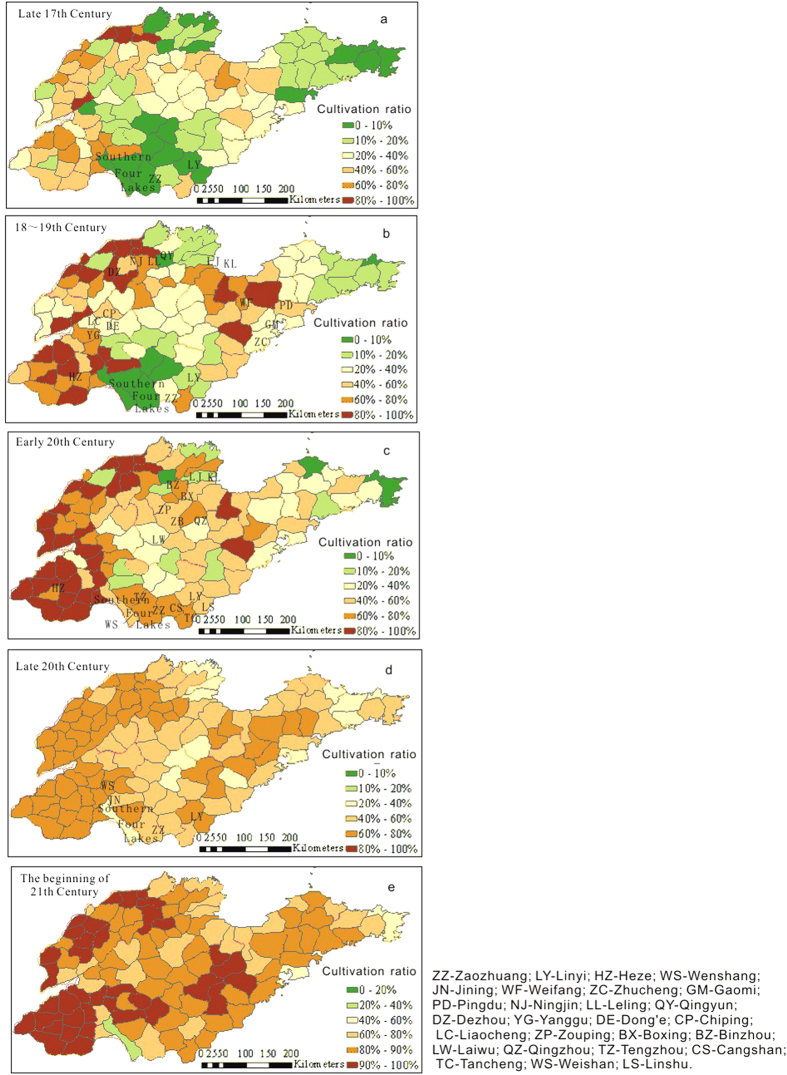
Change in the spatial distribution of cultivation ratios for Shandong over the past 300 years (map created using ARCGIS).

**Figure 9:**
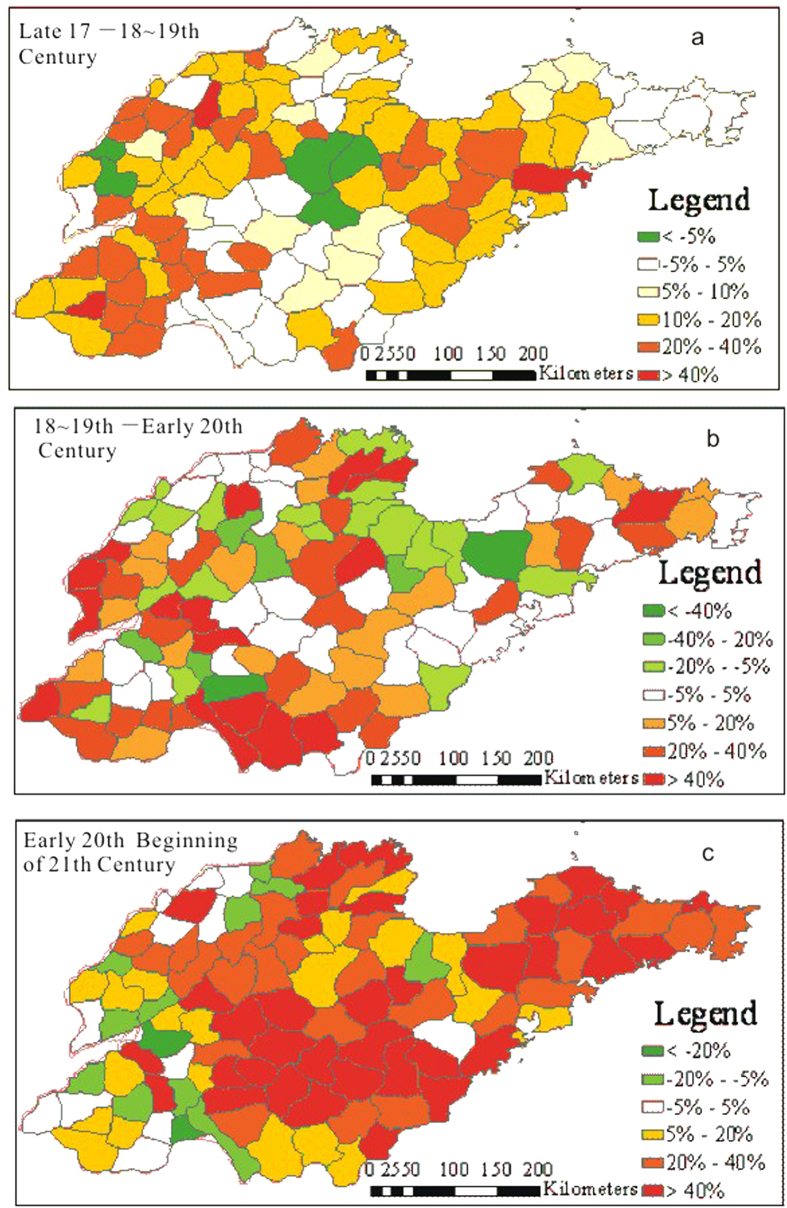
Change in the cultivation ratios in each county for Shandong in the 18^th^–21^st^ centuries (map created using ARCGIS).

The spatial distribution of cultivation ratios in the late 17^th^ century (Fig. 8a) shows that several agricultural centers with high cultivation ratios formed during that time, including southwestern Shandong, northwestern Shandong and the Jiao-lai Plain, where the cultivation ratios of most counties was greater than 40%. The cultivation ratios for the hilly areas of middle and southern Shandong, the hilly area of Jiaodong and the coastal lowland of northern Shandong are the lowest, with ratios of less than 10%; this result is particularly relevant in the Southern Four Lakes area and parts of the Zaozhuang-Linyi region, which have the lowest cultivation ratios in Shandong. These areas are located in the main flooding area of the Yellow River. During the 18^th^–19^th^ centuries, the spatial distribution of the cultivation ratios still retained the pattern of the late 17^th^ century (Fig. 8b). The cultivation ratios of the three agricultural areas increased unequally, by 10–40% (Fig. 9a). The hilly area of Jiaodong, the hilly area of middle and southern Shandong, and the coastal lowland of northern Shandong have been exploited to some extent.

The spatial distribution of cultivation ratios in Shandong during the early 20^th^ century (Fig. 8c) shows that the spatial pattern of cropland cover in Shandong at that time was similar to the pattern during the 18^th^–19^th^ centuries. The primary change was a rapid increase in the cultivation ratios for the Southern Four Lakes and Zaozhuang-Linyi areas, and this change was probably related to a rupture of the Yellow River dike followed by the Yellow River’s route changing northward. Several other regions increased or decreased differently, and these changes were distributed irregularly (Fig. 9b).

The distribution of cultivation ratios in the 1980 s (Fig. 8d) shows that the regional differences were becoming more subtle. The cultivation ratio for the three primary agricultural areas decreased to less than 80%. The cultivation ratios for most cities and counties were within 20%–80% at that time. The distribution of cultivation ratios in 2009 (Fig. 8e) shows that cultivation ratios for different regions generally increased. Most counties increased by 40%, compared with their early 20^th^ century ratios (Fig. 9c). Except for the relatively low cultivation ratio for the coastal lowlands, the regional differences in most areas were more subtle than the differences seen in the two previous periods.

### Analysis of cropland cover change and saturate extent

**Figure 10:**
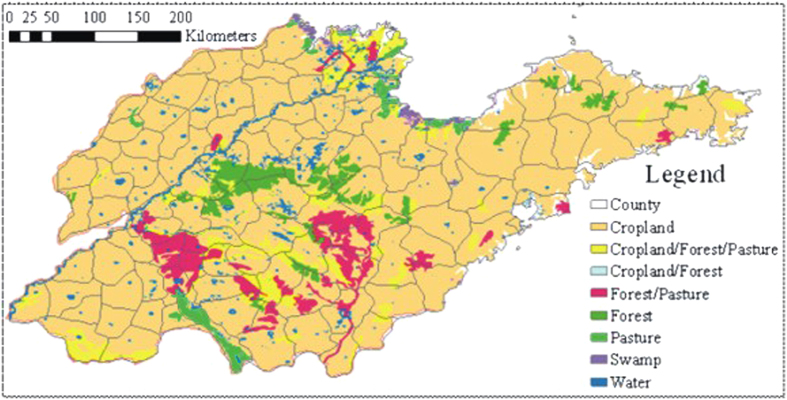
Land suitable type for Shandong (map created using ARCGIS).

We compared the map of Chinese land suitable type (Fig. 10) with the cultivation ratio map in the late 17^th^ century, the 18^th^–19^th^ centuries, and 1916. The cultivation ratios in southwestern Shandong, northwestern Shandong, and the Jiao-lai Plain, which were suitable for cultivation, are relatively higher. Most counties had cultivation ratios greater than 40%, and several counties had cultivation ratios greater than 60%. However, the cultivation ratios for the delta area of the Yellow River and in the coastal area of eastern Shandong during the 18^th^–19^th^ centuries improved compared with the ratios of the late 17^th^ century. The reclamation extent in Zibo, which is located to the north of middle and southern Shandong, and in the Zaozhuang and the Linyi area in middle and southern Shandong, noticeably, improved in 1916. However, the saturated extent of reclamation in the Heze area of southwestern Shandong decreased, and the saturated extent of reclamation in northwestern Shandong and the Jiao-lai Plain showed no clear trend.

The extent of cultivation is highest in southwestern Shandong. Cultivation ratios were greater than 40% in nine counties, greater than 60% in seven counties, and greater than 80% in two counties in the Heze area during the 18^th^–19^th^ centuries. Until 1916, the cultivation ratios for eight counties reached 60–80%, with only Wenshang County in the Jinin area reaching a ratio greater than 40%. The extent of cultivation in the Jiao-lai Plain and in northwestern Shandong is inferior to the extent of cultivation in southwestern Shandong. The cultivation ratios for Weifang, Zhucheng, Gaomi, and Pingdu in the Jiao-lai Plain during the 18^th^–19^th^ centuries were greater than 60%. The cultivation ratios for Ningjin, Leling, and Qingyun in northern Dezhou (northwestern Shandong) were greater than 80%. The cultivation ratios for Yanggu, Dong’e, and Chiping in southeastern Liaocheng area were greater than 60%. The cultivation ratios for the other regions were generally within 20%–60%. Based on the 1916 data, the saturation extent of cultivation in several counties in northwestern Shandong and in the Jiao-lai Plain increased, whereas the extent of saturation in several other counties decreased. The cultivation ratios for Zouping and Boxing in Binzhou City, Laiwu City and Qingzhou City in northern Shandong improved to greater than 40%.

The cultivation ratios in the Zaozhuang and Linyi areas, which were probably located in the flood area of the Yellow River during the 18^th^–19^th^ centuries, were the lowest, less than 10%. In 1916, Zaozhuang City, Tengzhou City, Cangshan, and Tancheng in the Linyi area reached ratios greater than 40%, and the cultivation ratios for Weishan, Linshu, and Linyi City were all greater than 20%. These areas were gradually cultivated as the Yellow River changed its route to the north.

The cultivation ratios were low in the hilly areas of middle and south Shandong, the Jiaodong Peninsula, and the coastal hilly area where the geography was not appropriate for reclamation. For example, the ratios for Kenli and Lijin, which were located in the modern delta area of the Yellow River during the 18^th^–19^th^ centuries, were mostly within 10–40%. In 1916, the modern delta area of the Yellow River was formed and cultivated, and the cultivation ratios for Kenli and Lijin reached 20–40%.

A comparison of a map of the cultivation ratios for Shandong during 2009 and a map of the land suitable types (Fig. 10) shows that land reclamation in Shandong became over-saturated in the 21^st^ century. Most suitable reclamation regions have been completely cultivated and hilly areas and coastal lowlands, which are not suitable for reclamation, have also been covered by cropland.

## Conclusions and Discussion

Generally speaking, an evaluation of the reconstruction method and the results of this study show that our results are reasonable and dependable. The conclusions of this study are as follows.

1. Compared with reconstruction studies by other scholars, the reconstructed data in this study represent the cropland area at every stage, and the time resolution is approximately at the century scale, which avoids the creation of a contingency error that is caused by the selection of one year of data as other scholars have done. The reconstructed croplands, which were developed by Ge *et al.* (2003), show three peak values, and these values may be related to the correction of the land and gazetteer records in every kingdom. In this study, we attempted to avoid this type of peak value; instead, we attempted to reconstruct cropland area changes of relatively stable stages. The revised data show that on a century scale, cropland area changes in Shandong do not show significant peaks, but increase exponentially. This trend is consistent with a natural increase in population and with cropland area changes in a relatively closed region. Altogether, these results on the trends and stages of croplands in Shandong are relatively consistent with the conclusions described by other researchers. In paticular, the resolution of reconstructed results has been greatly improved at a county level compared with the popular historical Chinese cropland cover data sets, such as Ge *et al.*^30^ and other research results shown in Fig. 6.
2. To study the spatial distribution of cultivation ratios in Shandong, this study used the cropland area data from a specific time period, which was collected from county gazetteers using a uniform standard and the same stage. These data embody the spatial differences in cultivation ratios for the province more accurately than data collected from provincial gazetteers or from the Fu (the modern equivalent of regional) gazetteers. The suitability of gazetteers in regional land use research has been discussed and tested in other studies^14^,^34^.
3. The spatial distribution of cultivation ratios from the 18^th^–19^th^ centuries to the early 20^th^ century shows no clear trend; however, during this time, the ratios of several counties increased and others decreased. The question remains whether the difference in cultivation ratios for these counties is caused by different data sources or whether the cropland areas experienced large fluctuations when the land reclamation was near saturation levels. In this study, we compared the revised gazetteer data from the early 20^th^ century with the revised 1916 statistical data from the Republic of China for the same areas and found that 60% of the data points in these overlapping areas were the same, with a 15% error or less. The two data sets were revised to account for tax discounts and exaggeration and cheating during reporting and were also normalized at the survey data level. The data from the gazetteers show larger cultivation ratios, which probably reflect inconsistencies in the time period selection and an increase in cropland during the beginning of the Republic of China. When dealing with data from the early 20^th^ century, prior to using gazetteer data or statistical data from the Republic of China, the data must be converted according to the regression equations that relate them; this step is needed to guarantee consistency with gazetteer data for the 17^th^–19^th^ centuries. Therefore, the reconstructed spatial distribution of cultivation ratios in the early 20^th^ century is reasonable. Compared with the changes in the 18^th^–19^th^ centuries, the changes in cropland area actually differed significantly during this period, which maybe a common characteristic when cropland is nearly saturated.
4. By comparing five spatial distribution maps of the cultivation ratios, it was determined that the cropland spatial distribution pattern in Shandong developed during the 18^th^–19^th^ centuries and reached saturation at the beginning of the 20^th^ century. This study showed that land that was suitable for cultivation was reclaimed more than land that was unsuitable for cultivation. Based on a comparison of two maps, one from the 1980 s and one from 2009 with a map of the beginning of the 21^st^ century, it is evident that the spatial differences in cultivation ratios have decreased. Comparing the 2009 map with the 1980 s map shows an increase in the cultivation ratios across the research area and also shows that the spatial differences decreased even further. This change in the distribution pattern of historical cropland is probably related to the changing route and flooding of the Yellow River, the introduction of sweet potatoes and corn, repair of water facilities and the exploitation of hilly areas and wetlands. The distribution patterns of the modern croplands are clearly affected by government land planning and by policies controlling cultivated land.
5. An analysis of the saturation extent of land reclamation showed that, at least before the early 20^th^ century, land reclamation was consistent with natural land appropriation. Except for a potential error in interpreting European satellite images (70% accuracy rate with 95% confidence)^29^, land reclamation in Shandong has been oversaturated since the beginning of the 21^st^ century; land appropriate for cultivation has been completely cultivated, and inappropriate land areas such as hills and coastal wetlands have also been converted to cropland. No additional land is available to sustain growth in population.

Furthermore, although the results are probably reliable, it should be noted that there are still uncertainties in the reconstruction estimates of cropland cover changes. On the one hand, original land records, which were gathered from gazetteers, deviate from the actual values due to the individual taxpayers’ intentions and land reports from the Qing dynasty and the Republic of China. On the other hand, the process of converting tax units for land to actual cropland area can also create deviations. The interpolation of data between different time periods according to their correlation and regression equations also introduced uncertainty into the results.

## Additional Information

**How to cite this article**: Ye, Y. *et al.* Reconstruction of cropland cover changes in the Shandong Province over the past 300 years. *Sci. Rep.* **5**, 13642; doi: 10.1038/srep13642 (2015).
